# Lack of association between sCTLA-4 levels in human plasma and common CTLA-4 polymorphisms

**DOI:** 10.1186/1477-5751-7-8

**Published:** 2008-11-12

**Authors:** Andrew Berry, Matt Tector, Martin K Oaks

**Affiliations:** 1 Transplant Research Laboratory, Aurora St. Luke's Medical Center, 2900 W. Oklahoma Ave., Milwaukee, WI, 53215, USA

## Abstract

**Background:**

Cytotoxic T lymphocyte antigen-4 (CTLA-4) is an important downregulatory molecule expressed on both T and B lymphocytes. Numerous population genetics studies have documented significant associations between autoimmune diseases and single nucleotide polymorphisms (SNPs) within and around the CTLA-4 region of chromosome 2 in man. Furthermore, circulating levels of a soluble form of CTLA-4 (sCTLA-4) have been reported in a variety of autoimmune mediated diseases. Despite these findings, the relationship between levels of sCTLA-4 protein, mRNA transcript levels, and SNPs within the CTLA-4 region have not been clearly defined. In order to further clarify this relationship, we have tested four different SNPs within the CTLA-4 region among subjects whom are negative (n = 53) versus positive (n = 28) for sCTLA-4.

**Results:**

Our data do not support a clear association between sCTLA-4 levels and any of the four SNPs tested.

**Conclusion:**

The variation in the SNPs tested does not appear to effect sCTLA-4 protein levels, despite reports that they affect sCTLA-4 mRNA.

## Background

Human chromosome region 2q33 contains three genes known to be involved in immune regulation [1]. Two of these genes appear to positively regulate immune responses. These are the CD28 receptor gene and the inducible co-stimulator (ICOS) gene. A third gene appears to be a negative regulator of T cell activation; namely, CTLA-4 [2,3]. It is thus not surprising that genetic variation within this region is implicated in engendering susceptibility to autoimmune disease. The CTLA-4 gene yields at least two major mRNA transcripts in man [4]. One encodes a transmembrane protein that plays an important role in downregulating T lymphocyte activation. The other transcript encodes what appears to be a soluble form of CTLA-4 that lacks a transmembrane domain, so the protein product should be found in the extracellular space including blood plasma [5]. We, [6] and others [7] have identified immunoreactive material in human plasma that appears to represent the sCTLA-4 protein. Extensive population genetics studies have suggested associations between SNPs in and around the CTLA-4 locus on chromosome 2 in man and the presence of autoimmune disease [8]. The first of these reports was made by Yanagawa et al [9] in 1995, who found a significant association between variation in the (AT) dinucleotide repeat within the 3'-untranslated region of the CTLA-4 gene and the presence of Grave's disease. Subsequent to these findings, many others have reported associations between SNPs within and around the CLTA-4 region and rheumatoid arthritis [10,11], celiac disease [12-14], type I diabetes, [15], myasthenia gravis [16,17] and autoimmune pancreatitis [18]. At the protein level, a variety of studies have implicated elevated levels of the sCTLA-4 protein in the plasma of patients with a variety of immunologically mediated diseases including autoimmune thyroid disease [6,19], systemic lupus erythematosus [20] cutaneous systemic sclerosis [21], allergic asthma [22,23], psoriasis vulgaris [24], and autoimmune pancreatitis [25].

In a landmark study of SNP analysis within a 330 kb region of chromosome 2q33 containing CD28, CTLA-4 and the ICOS gene regions in type I diabetics, Ueda et al [15] implicated the CT60 SNP (rs3087243) as playing an important role in the risk of development of diabetes. Interestingly, the "G" susceptibility allele appeared to be related to decreased levels of the sCTLA-4 mRNA relative to those of the full-length (transmembrane encoding) transcript. Subsequent to this report, a SNP within the ICOS gene region (IVS+173, also on chromosome 2q33), was reported to influence alternate splicing of CTLA-4 isoforms [26].

Despite the interesting associations between genetic variation near these immunoregulatory gene regions, mRNA transcript levels, and blood levels of sCTLA-4, a clear functional relationship between them and the pathogenesis of autoimmune disease have not been elucidated. We speculated that if the CT60 SNP or other SNPs within and in proximity of the CTLA-4 gene region were associated with changes in sCTLA-4 mRNA levels, the same SNPs might also be associated with changes in the amount of sCTLA-4 protein in blood plasma. To this end, we selected both positive and negative (undetectable) plasma samples for sCTLA-4 and performed SNP analysis for four commonly tested SNPs within and around the CTLA-4 region. We found no statistically significant differences in observed vs. expected genotypic frequencies for these SNPs when comparing positive vs. negative blood levels of sCTLA-4. Thus, our data do not support a relationship between these commonly tested SNPS and circulating levels of sCTLA-4 in the presence or absence of autoimmune disease.

## Methods

### Study Population

The sample set consisted of 81 serum samples from patients with a variety of autoimmune disease (n = 54) or normal adult volunteers without a history of autoimmune disease (n = 27). They were segregated without reference to disease status on the basis of the presence or absence of elevated levels of sCTLA-4 as described below. Blood samples were obtained following informed consent, and the study was done under the oversight of our local Institutional Review Board.

### Laboratory Analysis

Sera from human subjects were tested in a sandwich ELISA for sCTLA-4 as previously described [6]. Samples were categorized as positive or negative for sCTLA-4 based upon a cutoff optical density of 2.5 fold increase over the OD450 nm observed when tested against an irrelevant capture antibody. In general, this corresponded to sCTLA-4 levels on the order of > 10 ng/ml as defined by commercially available test kits. Triplicate determinations were made with both anti-CTLA4 and irrelevant capture antibodies.

SNP genotyping was performed on DNA samples obtained from white blood cell pellets using the Qiagen mini kit (Chatsworth, CA) as described in the manufacturers instructions. Polymerase chain reaction was used to amplify DNA fragments including SNPs. PCR products were digested with appropriate restriction enzymes and subjected to standard agarose gel electrophoresis for analysis.

CT60 (rs3087243) genotyping was performed as described in Vigano et al. [27]. The + 49 A/G (rs231775) and -318 (rs5742909) SNPs were determined as described by Harbo et al. [28]. IVS1+173 (rs10932029) T/C genotyping was performed as described by Hunt et al. [14].

### Statistical Analysis

The Freeman-Halton Extension of the Fisher Exact Test (two tailed) was used for comparison of the distribution of observed genotypes for each polymorphism when compared to expected genotypes based upon previously published allele frequencies. The following allele frequencies were used to calculate expected genotypic frequencies: CT60 A = 0.477, G = 0.523; +49A/G A = 0.642, G = 0.358; -318 C = 0.91, T = 0.09; IVS+173 T = 0.86, C = 0.14. Allele frequencies are from Ueda et al. [15], with the exception of IVS+173, which is from Haimila et al [29]. Expected frequencies were calculated based on the Hardy-Weinberg formula.

## Results and Discussion

We tested 28 individuals who were positive and 53 who were negative for sCTLA-4 in blood plasma for the purpose of determining whether there was an association with common SNPs within the CTLA-4 and ICOS regions of human chromosome 2q33. No evidence of an association between levels of sCTLA-4 and SNP genotypes were found (Table 1.). Furthermore, there were no statistically significant differences in absolute allele counts between positive and negative sera (data not shown). Although the number of samples is rather small, there were no clear correlations between absolute levels of sCTLA-4 protein and SNP genotypes.

**Table 1:** Distribution of genotypes of chromosome 2 SNPs among sCTLA-4 positive and negative patients.

		sCTLA-4	Pos (N = 28)	sCTLA-4	Neg (N = 53)
			
Polymorphisms	**Genotypes**	**Observed**	**Expected**	**Observed**	**Expected**
	AA	8	6	12	12
CT60	AG	12	14	28	26
	GG	8	8	13	15
	AA	11	11	26	22
					
+49 A/G	AG	14	13	21	24
	GG	3	4	6	7
	CC	23	23	50	44
					
-318 C > T	CT	4	5	2	8
	TT	1	< 1	1	< 1
	TT	20	20	38	38
					
IVS1 +173 T/C	TC	8	8	20	15
	CC	< 1	< 1	< 1	< 1

There were no statistical differences between observed and expected genotype frequencies among either patients positive (Pos) or negative (Neg) for sCTLA-4 as determined by ELISA. Data are genotype counts. Expected counts were calculated using the Hardy-Weinberg formula based on previously published gene frequencies (15,29). N = number of subjects in each group. See text for definitions of polymorphisms.

Our data confirm and extend the findings of Purohit and co-workers [30], who reported a lack of association between CT60 genotype and sCTLA-4 levels. On the other hand, our findings appear to be at odds with the speculation that the CTLA-4 CT60-A/G SNP may determine the alternate splicing and production of the sCTLA-4 mRNA [15]. In the Ueda model, the CT60-G susceptibility allele appears to produce lower relative amounts of the sCTLA-4 mRNA; thus, one would expect that subjects at risk for autoimmune disease to have reduced levels of sCTLA-4 protein. It seems paradoxical given that lower levels of CTLA-4 message are present in susceptible individuals whereas higher levels of sCTLA-4 protein are observed in plasma of individuals with autoimmune disease. Possible explanations for the appearance of this discrepancy may include the possibility that there is no direct relationship between message levels at the cellular level and circulating protein in plasma. For example, elevated circulating sCTLA-4 levels may simply be due to increased half-life and/or decreased turnover of protein despite increased levels of synthesis. Also, it is possible that lower levels of sCTLA-4 message reflect a feedback regulatory loop in which mRNA levels are reduced in the face of higher levels of sCTLA-4 protein. Finally, it is possible that immunoreactive CTLA-4 material detected in human serum is not the direct gene product of the sCTLA-4 mRNA transcript. While our lab [5,6] has previously reported the presence of a novel epitope (which is predicted to arise from a frameshift due to alternate splicing) in immunoprecipitates from CTLA-4 monoclonal antibodies, only a minority of the material from these immunoprecipitation experiments is of the predicted molecular mass of the sCTLA-4 monomer (23 kDa). Thus, it is possible that ELISA based assays for circulating CTLA-4 levels cannot distinguish sCTLA-4 monomer produced directly by the sCTLA-4 transcript within a heterogeneous population of CTLA-4 immunoreactive material derived from other sources, such as that derived from proteolytic cleavage from cells that express the transmembrane protein. There are numerous examples of soluble receptors that are derived from such a mechanism including many of the members of the tumor necrosis factor receptor family as well as other cytokine receptors and adhesion molecules [reviewed in [31]]. Despite the finding that the IVS+173 SNP appears to affect the relative level of sCTLA-4 mRNA [26], our data suggest that the same SNP does not directly control circulating levels of sCTLA-4 protein. In any case, the precise mechanism that controls levels of the sCTLA-4 transcript and sCTLA-4 immunoreactive material needs to be further investigated, but there does not appear to be a simple relationship between the SNPs that are the object of study in this report and the sCTLA-4 protein.

## Abbreviations

CTLA-4: Cytotoxic T-lymphocyte antigen-4; sCTLA-4: soluble CTLA-4; SNP: single nucleotide polymorphism; rs: reference SNP (from NCBI dbSNP database: ).

## Competing interests

The authors declare that they have no competing interests.

## Authors' contributions

MKO wrote the manuscript, participated in designing the study, and performed statistical analysis. AB performed SNP testing, data organization, and analysis. MT participated in designing the study and drafting of the manuscript.
